# Hierarchical cooperation of transcription factors from integration analysis of DNA sequences, ChIP-Seq and ChIA-PET data

**DOI:** 10.1186/s12864-019-5535-2

**Published:** 2019-05-08

**Authors:** Ruimin Wang, Yunlong Wang, Xueying Zhang, Yaliang Zhang, Xiaoyong Du, Yaping Fang, Guoliang Li

**Affiliations:** 1 Agricultural Bioinformatics Key Laboratory of Hubei Province, Wuhan, 430070 China; 2 Huazhong Agricultural University, Wuhan, 430070 China; 3 College of Informatics, Huazhong Agricultural University, Wuhan, 430070 China

**Keywords:** Chromatin loop, Transcription factor cooperation, Hierarchy and dynamics, Enhancer, Promoter

## Abstract

**Background:**

Chromosomal architecture, which is constituted by chromatin loops, plays an important role in cellular functions. Gene expression and cell identity can be regulated by the chromatin loop, which is formed by proximal or distal enhancers and promoters in linear DNA (1D). Enhancers and promoters are fundamental non-coding elements enriched with transcription factors (TFs) to form chromatin loops. However, the specific cooperation of TFs involved in forming chromatin loops is not fully understood.

**Results:**

Here, we proposed a method for investigating the cooperation of TFs in four cell lines by the integrative analysis of DNA sequences, ChIP-Seq and ChIA-PET data. Results demonstrate that the interaction of enhancers and promoters is a hierarchical and dynamic complex process with cooperative interactions of different TFs synergistically regulating gene expression and chromatin structure. The TF cooperation involved in maintaining and regulating the chromatin loop of cells can be regulated by epigenetic factors, such as other TFs and DNA methylation.

**Conclusions:**

Such cooperation among TFs provides the potential features that can affect chromatin’s 3D architecture in cells. The regulation of chromatin 3D organization and gene expression is a complex process associated with the hierarchical and dynamic prosperities of TFs.

**Electronic supplementary material:**

The online version of this article (10.1186/s12864-019-5535-2) contains supplementary material, which is available to authorized users.

## Background

The spatial structure of the genome is important to cellular functions, such as gene expression and regulation, cell differentiation and identity, and tissue development [1–3]. Linear DNA, which is 2 m long in one human cell, is highly packed into chromatin and adapts to the size of the cell nucleus. With the development of high-throughput sequencing technologies, new methods, such as high-throughput chromosome conformation capture (Hi-C) [4], in situ Hi-C [5], have been developed and applied to study the spatial organization of chromatin in various human cells. The structure of genomic DNA can be compartmentalized into four hierarchical structures [6–8] with different resolutions: chromosome territories (50–250 MB bases), A/B compartments (~ 5 MB bases), topologically associated domains (TADs) (~ 1 MB) or sub-TADs (0.1–1 MB), and chromatin loops (5–300 kb) [7]. Among these structures, the chromatin loop is the architectural basis of other higher structures. The chromatin loop can bring distal regulatory elements, such as enhancers in linear DNA, to the promoters of target genes in 3D space. As an example, recently, Chen and Levo et al. [9] reported that gene activation in *Drosophila* embryos is required for the sustained proximity of enhancer to its target promoters. Although the chromatin loop has an important role in gene regulation and disease [10], the specific mechanism, especially the involved molecules, such as proteins, and the cooperation of transcription factors (TFs) participating in the chromatin loop, remains poorly understood.

Genome-wide profiling of the TF binding sites is extensively studied across linear genomic DNA [11–13]. For example, information on many TF binding sites of various cells is deposited in the Encyclopedia of DNA Elements (ENCODE) project at the UCSC [11]. The cooperation between regulatory factors, such as TFs, histones, and DNA-associated proteins, is investigated by ChromNet using the public ENCODE ChIP-Seq (Chromatin Immunoprecipitation Sequencing) datasets [14]. Different combinations of TFs can result in various expression types in different tissues and specific expression types in different cell types and stages [13]. Although these works reported many instances of cooperation between different TFs, only linear DNA sequence information and the co-localization of different proteins across the linear genome were considered. However, substantial evidence indicates that different TFs can cooperate in 3D space and mediate interactions between distant sequences in the linear genome [5, 10, 15].

Recently, the molecular mechanisms and proteins in chromatin interactions have been studied. In situ Hi-C [5] can produce a one kilo-base resolution map of the global human 3D chromosome, and the results show that CTCF plays a predominant role in loop anchors. Further work using promoter capture Hi-C [16] demonstrated that the interacting regions between enhancers and promoters, especially long-range interactions, are important to cell lineage and human diseases [17]. Moreover, a previous study revealed that TFs mediate long-range enhancer–promoter interactions [18]. On the basis of 5 kb-resolution in situ Hi-C datasets and incorporation of public ChIP-Seq data, DBPNet [19] has been developed to identify protein combinations that mediate chromatin loops. A 1 kb resolution is relatively high for traditional Hi-C data but still low for TF binding sites, which are usually 5 to 25 base pairs long. These works also revealed the global map of human chromosome 3D information with a low resolution for TF DNA binding sites.

To obtain a nucleotide resolution map of cells, scientists have developed the ChIA-PET (Chromatin Interaction Analysis by Paired-End Tag sequencing) [10, 20] to search for the chromatin interactions associated with particular proteins, such as CTCF and RNA polymerase II. The ChIA-PET method incorporates ChIP-based enrichment, chromatin proximity ligation, and paired-end tags to determine chromatin interactions across the whole 3D genome. Our previous work revealed that CTCF, together with RAD21 and SMC3, mediates the 3D genome architecture of cells [10]. Our method provides an alternative means to show the TF binding sites in nucleotide resolution and 3D mode. Recently, 3CPET has been developed to search for the co-factor complexes in chromatin interactions from ChIA-PET data [21]. This work used the proximal sequence information between DNA–DNA contacts in 3D space and calculated the enrichment between different TF binding sites across the 3D space of DNA contacts. However, few studies have explored the hierarchical and dynamics analysis of TF cooperation using both ChIA-PET and ChIP-Seq data. There is a great need to develop a method for systematically evaluating the role of different combinatorial TFs involved in chromatin interactions that uses various data, such as 3D ChIA-PET and ChIP-Seq linear information.

In the present study, we provided the HidPET (Hierarchical and Dynamics Analysis of TF Cooperation with ChIA-PET and ChIP-Seq Data) method to study the hierarchy and dynamics of TF cooperation by integrating ChIP-Seq and ChIA-PET datasets. This method mainly focuses on enhancer–promoter interactions, which play a dominant role in chromatin interaction. The networks are constructed by using the enrichment information of the 3D chromatin data of ChIA-PET and the 1D linear genomic data of ChIP-Seq. Then, the networks are fused with the additional protein–protein interactions (PPIs). The hierarchy and local network parameters are analyzed across four cell lines. Hierarchical structure, community and clique analysis revealed the hierarchical and dynamic features of synergistic cooperative TF interactions in regulating gene expressions and chromatin 3D architecture.

## Results

### Promoter–enhancer interaction analysis

**Fig. 1:**
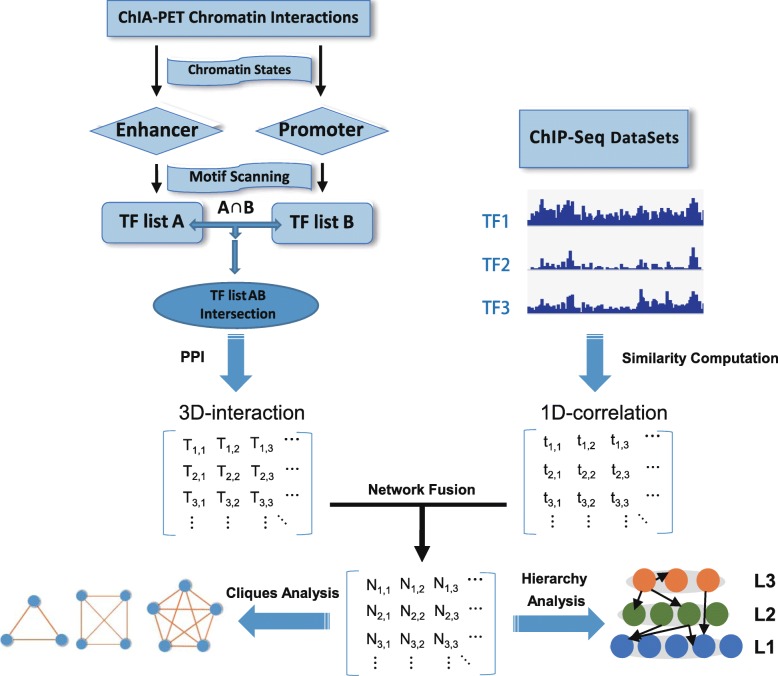
Schematic of the HidPET pipeline. TF represents the transcription factors. L represents the hierarchy of the TF network. “1D similarity” refers to the matrix for the network with TF similarities from the ChIP-Seq datasets in linear DNA sequence. “3D similarity” is the network with protein–protein interactions of TFs that are shared in both enhancers and promoters. PPI represents protein–protein interactions. Such PPI interactions include the intersection between those from STRING and BioGRID databases

We developed the HidPET method to combine ChIA-PET and ChIP-Seq data with PPI data to systematically study the hierarchy and dynamics of TFs in four cell lines. The flow chart of the HidPET method is presented in Fig. 1. The ChIP-Seq data of 237 human TFs from the ReMap database [22] are used to calculate the similarities between different pairs of TFs along the 1D linear genome as the 1D similarity matrix (See Methods). ChIA-PET datasets for four human cell lines, such as the human immortalized myelogenous leukemia line (K562), human breast adenocarcinoma cell line (MCF7), human umbilical vein endothelial cell line (HUVEC), and B lymphocyte cell line (GM12878), are downloaded from public databases [10, 15, 23–25] (Additional file 1) to generate a 3D similarity matrix.

In general, the genomic segments of chromatin interactions are enriched in promoters and enhancers. Enhancers extensively interact with promoters, which form loops and regulate the expressions of distant or proximal genes [18]. In our method, the anchors of loops are annotated with chromatin states from the same cell line and the loops included both enhancers and promoters were selected for further study. The anchor regions of enhancers and promoters were scanned with the position weight matrices (PWMs) of 980 TFs with known motifs in the database of the predrem [26]. Then, we obtain all the TFs appearing at least once in the enhancer anchors as TF list A and all the TFs appear at least once in the promoter anchors as TF list B. The shared PPIs from both STRING and BioGRID are overlapped with the intersection of the TF list A and B and kept as 3D similarity matrix. The similarity between TFs from the ChIP-Seq datasets was calculated using a method from IntervalStats [27] as 1D similarity matrix. The 3D and 1D similarity matrices are fused depending on their shared TFs with the similarity network fusion method [28], and the interactions are changed to the connection network (as fused matrix). Finally, the fused matrix is used to calculate the hierarchical and dynamic properties of TF combinations (See Methods).

### Network community construction

**Fig. 2:**
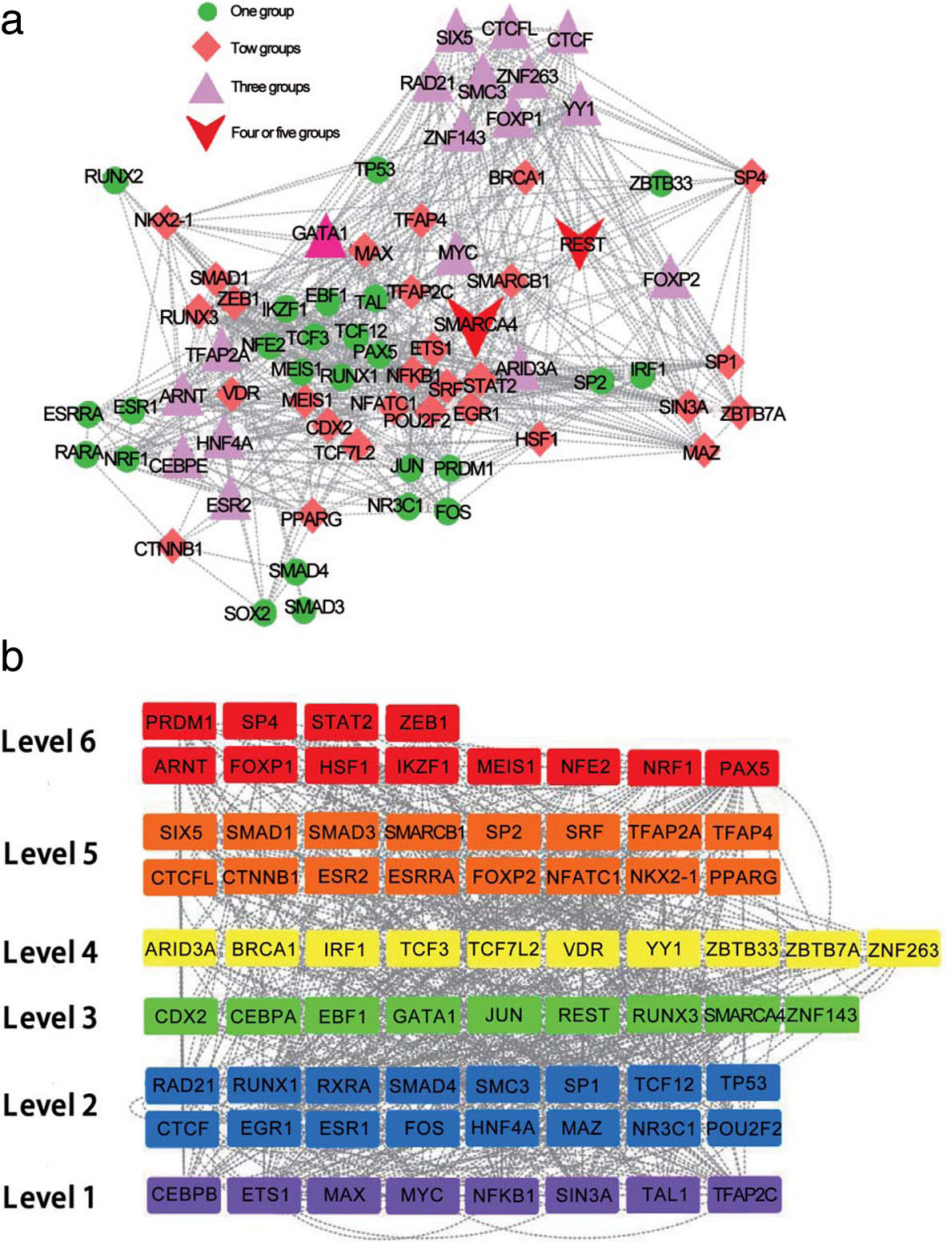
Communities and hierarchical structures of the GM12878 TF network. **a** Communities of the GM12878 TF network. The green circle represents TFs in one group. The light-coral diamond represents TFs in two groups. The pink triangle represents the TFs in three groups. The red V represents the TFs in more than four groups. **b** Hierarchical structures of the GM12878 TF network. Each level is represented in different colors. The regulation is represented by a dash line

A community of PPI is a candidate functional module [29, 30]. The fused matrix of 1D and 3D matrices for TFs is used to identify the community structures. In the fused matrix, each set of TFs in a community is densely and sparsely connected between communities. Community structures of a network can be divided into non-overlapping communities, where a given node can only be included in one group, and overlapping communities, where a given node may be included in multiple groups. In biology, previous studies indicated that one protein can participate in several regulatory pathways [29, 31] with different roles; an example is YY1 (Yin Yang 1) [31], which is involved in activating or repressing gene transcription. Here, we adopt the fused network of GM12878 as an example to illustrate the network communities of TFs and applied ClusterONE [29] (Clustering with Overlapping Neighborhood Expansion) to identify the overlapping communities for the fused matrix. Nine groups are calculated, and the specific list of TFs in each community is listed in Additional file 2. The global network of GM12878 for 73 TFs is given in Fig. 2a. Results indicate that 65.8% (48/73) TFs participate in two or more communities. This finding implies that a TF may operate in two or more pathways and has multiple functional roles in gene regulation. However, most of the TFs (97.2%, 71/73) are grouped in less than four communities. Only SMARCA4 and REST TFs participate in more than four groups. SMARCA4 is a part of the ATP-dependent chromatin remodeling complex SNF/SWI and can regulate gene transcription by altering the chromatin structure around the genes [32]. SMARCA4 is involved in the pathway of DNA damage and translational control; the TF can bind to chromatin and has a transcription coactivator activity [33]. REST is a transcriptional repressor that regulates gene expression by binding to a repressor element [34] and is related to the chromatin organization pathway. REST can regulate the SMARCA4 gene and is linked to schizophrenia [35]. Figure 2a also indicates that 34.2% (25/73) of the TFs are grouped in one community; this finding implies that these TFs may be functional in a specific pathway. Moreover, 38.4% (28/73) and 24.7% (18/73) of the TFs can simultaneously participate in two and three communities, respectively. These TFs may have multiple roles in gene regulation. For example, several chromatin structure-related TFs, such as CTCF, RAD21, SMC3, and YY1, are involved in three groups. Figure 2a shows that the TFs from different communities are connected. Most of the TFs rarely independently regulate gene expression but usually work synergistically with other TFs from different communities.

### Construction of hierarchical network

The directed biological network can be compared with the “chain-of-command” structures in social networks [36]. A previous work proposed a model with three hierarchical levels (top, core, and bottom) for representing the network architecture of a yeast transcription network [37]. They suggested [37] that the top TFs are responsible for conditional changes while the core and the bottom TFs are responsible for information propagation with noise minimization. However, the model cannot calculate the number of levels and the position of ambiguity nodes with the probability to remain at each level. Recently, the hierarchy score maximization (HSM) algorithm [38] has been proposed to calculate the hierarchical structure capable of overcoming the problems of the number of levels and the ambiguous nodes. A simulated annealing approach was used to calculate the number of hierarchical levels of a directed network by the HSM algorithm, and the probability is calculated for any ambiguity node to be at each level [38]. To quantify the hierarchical structure of the fused TF matrix, we calculate for the corrected hierarchical score by using the HSM algorithm. For the fused TF matrix, the direction between two TFs retains the same direction as that in the 1D matrix. One pair of TFs without direction in the 1D matrix is removed, and the final matrix has 71 TFs with directions for the GM12878 cell. The number of levels is optimized from 2 to 8 with an interval of 1 by calculating for the corrected hierarchy score. A higher corrected hierarchy score indicates a higher likeliness of the level in question. For the GM12878 cell, the 6-level possesses the largest corrected hierarchy score (Additional file 3: Figure S1a) and is selected as the final hierarchical structural level. The global hierarchical structure of TFs in the GM12878 cell is given in Fig. 2b. The figure shows that the chromosome architecture proteins, such as CTCF, RAD21, and SMC3, are in the same level 2. Figure 2b also indicates many interactions across different levels. The 2nd and 5th levels have the largest numbers of TFs (16). To quantify the trend of connections between different levels, we calculate for the number of links between different levels. The ratio of the number of links between different levels to the largest theoretical number of links between different levels is also calculated (Additional file 3: Figure S1b). The results indicate that the 1st, 2nd, 3rd, and 4th levels tend to be enriched links with other levels. The number of links for the TFs from different levels is higher than that of the links for the TFs from the same levels. For the 6th, 5th, and 4th levels, the larger ratio tends to be connected with the 1st and 3rd levels. This result means that the TFs tend to be cross-talked from different levels, and the high levels tend to be linked to the low levels. For the links of TFs within the same level, the 1st level has the largest ratio of interactions with the other TFs. These results reveal the presence of a hierarchical structure organization in the TF network, and the TFs from different levels tend to be cross-linked. We further mapped the TFs from communities to different levels. The results indicate that all nine communities are distributed in the six levels. This result means that a community tends to be constituted by TFs derived from different levels.

In addition, because the TFs do not arise simultaneously, they generally occur through the re-organization of pre-existing genes or de novo [39]. De novo genes are also believed to emerge through the evolution of lineages [39]. A recent study has systematically investigated the differences of gene expressions in tumors on the basis of the evolutionary history of genes from 16 clades, which range from cellular organisms (phylostratum 1) to *Homo sapiens* (phylostratum 16) [40]. To understand the evolutionary history difference of all these TFs from 16 clades, we grouped the TFs into two classes, namely, before and after the Bilateria clade (phylostratum 6). The percentage of TFs from different levels in such two classes are given (Additional file 3: Figure S2). The results reveal that the TFs in each level consist of both classes. The TFs in levels 1 and 2 tend to be enriched before the Bilateria clade (phylostratum 6), and the TFs in levels 5 and 6 tend to be enriched in and after the Bilateria clade. This finding suggests that the new TFs tend to be enriched in higher levels and may regulate the interactions of enhancers and promoters.

### Maximal clique analysis

**Fig. 3:**
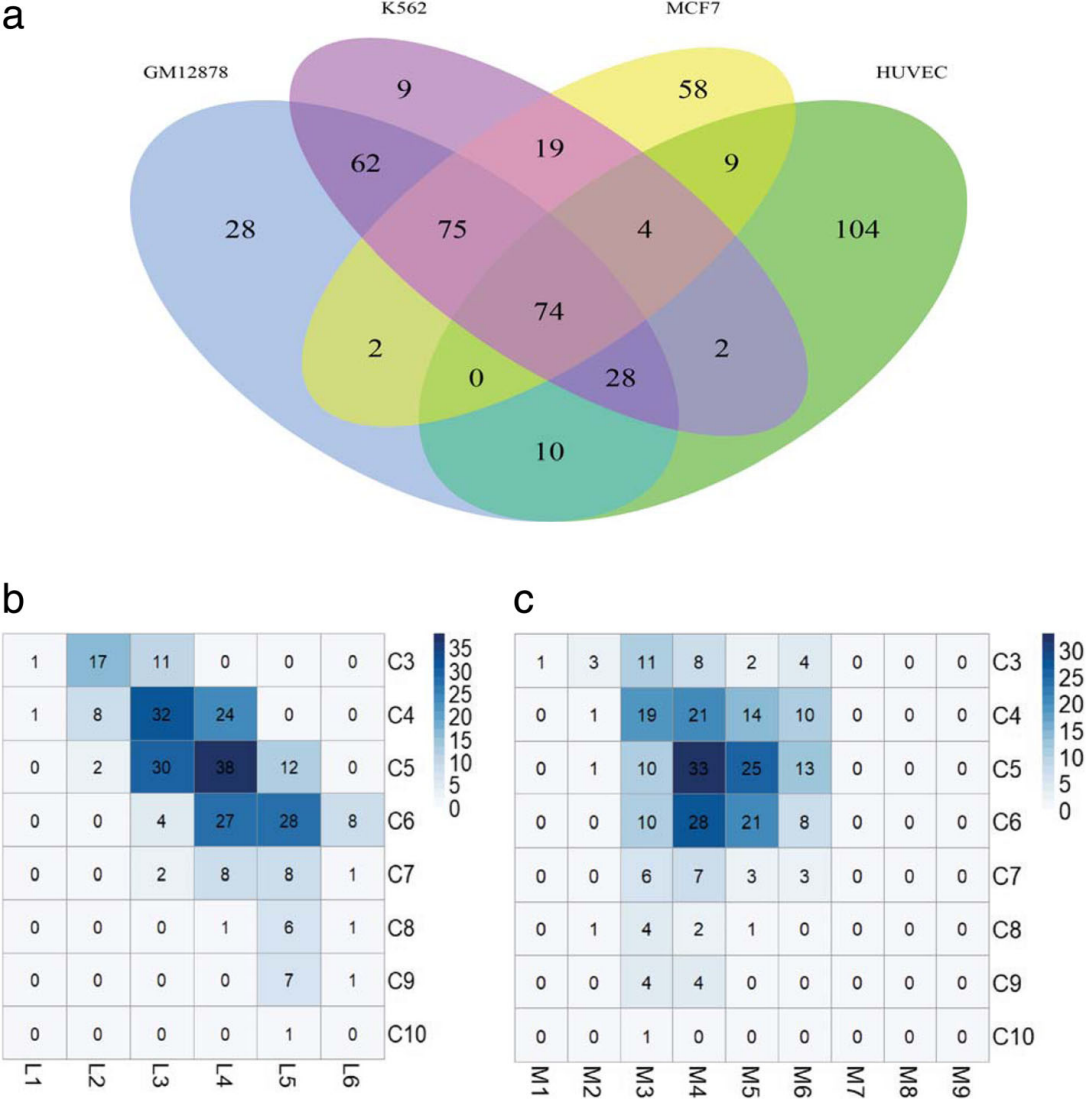
Relationship of maximal cliques, communities, and hierarchical levels. **a** Venn diagram of maximal cliques in four cell lines. **b** Clique distribution in different levels for the GM12878 cell. Here, Cn represents the clique with n TFs in the corresponding clique. Ln represents the level n in the hierarchy. **c** Clique distribution in different communities. Mn represents the community

Although TFs have a community structure and hierarchical structure to synergistically work together, the specific cooperation between TFs is not fully understood. To further analyze the cooperation between TFs, we calculate for the maximal cliques for the fused matrix of TFs in different cell types. The clique of a network is a fully connected sub-graph, which is a basic topological module of a network. In this work, we apply the MClique method in Cytoscape [41] to detect the maximal cliques. The number of TFs in the maximal clique is optimized from 3 to 10 with an interval of 1. The Venn diagram of the maximal cliques in the GM12878, K562, HUVEC, and MCF7 cell lines is presented (Fig. 3a). The specific TF in each maximal clique for the four cell lines is listed in Additional file 4. The total numbers of maximal cliques for GM12878, K562, HUVEC, and MCF7 are 279, 273, 231, and 241, respectively. Most cliques are shared by two or more cell lines, and the number of maximal cliques shared by all four cell lines is 74. This result suggests that these shared cliques may have similar functions in different cell types, and the shared cliques may be involved in the basic regulated pathway, which is conserved in cells and tissues. The numbers of cell line-specific cliques in each cell line are 28, 9, 104, and 58 for GM12878, K562, HUVEC, and MCF7, respectively. This result means that the specific cliques may execute cell specific functions that can shape the specific cell.

To further characterize the synergistic feature of TFs in a given clique, we calculate for the Spearman’s rank correlation coefficients of the TFs’ expression. For each cell line, the corresponding expression levels of these TFs are collected from the RNA-Seq data deposited in the ENCODE project. The Spearman‘s rank correlation coefficient is calculated for the expression of all pairs of TFs within the same maximal clique. The same procedure is applied to all pairs of TFs that are not in the same maximal clique. The distribution of Spearman’s rank correlation coefficients is shown in a violin plot (Additional file 3: Figure S3). The Spearman’s rank correlation coefficient of the TFs within the same maximal clique is significantly larger than that of the TFs randomly selected by Wilcoxon test. The *p*-values for the GM12878, K562, HUVEC, and MCF7 cell lines are 0.00265, 2.2e-16, 2.2e-16, and 0.00125, respectively. This result means that the corresponding genes of the TFs in the maximal clique tend to be co-regulated and work synergistically.

To analyze the hierarchical and community properties of maximal cliques, we calculate the distribution of TFs in maximal cliques across different hierarchies and communities. The TFs in each clique are mapped to the hierarchical structures and the communities. The results are presented in Fig. 3b. The cliques are represented with Cn, where C refers to clique and n refers to the number of TFs in such clique. Because the number of TFs in the cliques ranges from 3 to 10, the vertical axis ranges from C3 to C10.

For the hierarchical structures of TFs in cliques, the TFs in a given clique tend to lie across several levels of hierarchical structures. The cliques with four and five TFs tend to operate synergistically working across three or four levels. However, the cliques with more than five TFs tend to synergistically work across four or five levels. Meanwhile, the cliques with three TFs tend to synergistically function in two or three levels. Few cliques execute their function within only one level. These observations reveal that the TFs of a maximal clique usually originate from different levels in hierarchical structure and tend to cooperate in executing functions.

For the communities of TFs in cliques, a given TF in a clique can participate in multiple communities. Because a TF can participate in two or more pathways, TFs may be grouped into several communities. Figure 3c shows that most TFs in cliques are involved across five or six communities and enriched in three, four, or five communities. The cliques with smaller sizes tend to participate in a greater number of communities, and the larger cliques tend to be restricted within a community. In particular, the cliques with three TFs participate in six communities, and the cliques with four TFs have a similar result. This observation indicates that a TF tends to form multiple cliques across multiple communities. Moreover, TFs in a clique tend to participate across different communities, and communities can share the same clique in different cell types.

### Pattern analysis of cliques in cells

**Fig. 4:**
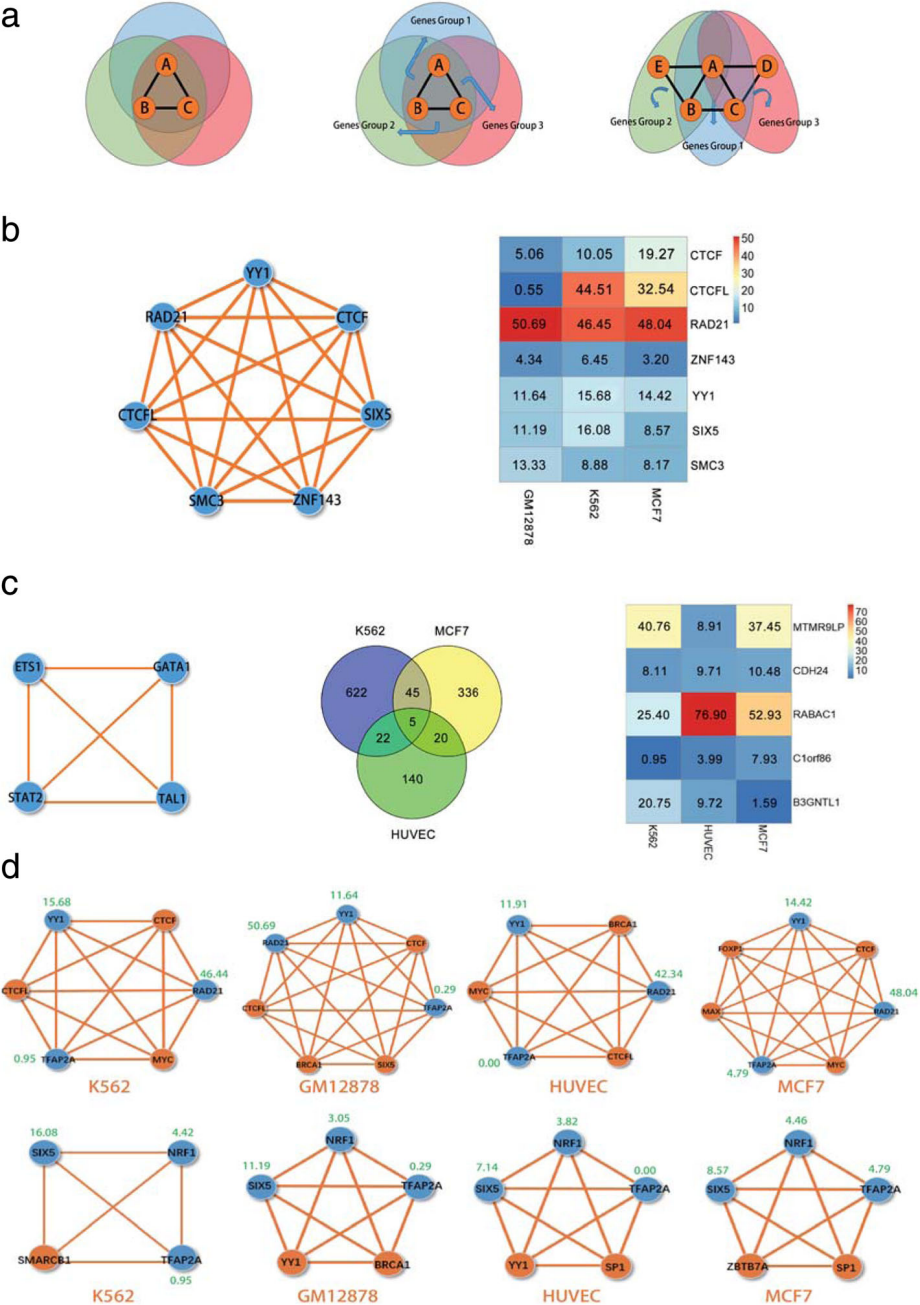
Schematic of the cliques and the corresponding gene expression levels in different cells. **a** Schematic of the cliques in different cell lines. **a**, **b**, and **c** represent a clique with three TFs. The Cyan, green, and magenta circles represent different cell lines. **b** Examples of shared cliques with seven TFs, Venn diagram of the number of overlapped regulated genes by the clique and gene expressions for these seven TFs in GM12878, K562, and MCF7 cells. **c** Clique and gene expression in K562, MCF7, and HUVEC cells. Clique with four TFs shared by K562, MCF7, and HUVEC cells. Venn diagram of the number of overlapped regulated genes by the clique in K562, MCF7, and HUVEC cells. The expression of the regulated genes by the clique in K562, MCF7, and HUVEC cells. **d** Two examples of cliques with RAD21, YY1, and TFAP2A and cliques with SIX5, NRF1, and TFAP2A shared by K562, GM12878, HUVEC, and MCF7. The shared TFs in cliques are colored blue, and the other TFs in cliques are colored orange. The expression levels of the shared TFs are labeled with the corresponding genes

To further analyze the cooperation of TFs in a given clique shared by different cell types, we investigate the patterns of cliques of the shared TFs and group these patterns into three different classes. The patterns of cliques are illustrated in a schematic (Fig. 4).

In one situation, a clique is shared by different cell types. This situation can be further sub-divided into two patterns. In the first pattern, although the target genes of a shared clique are nearly the same in different cell types, the expression levels of the corresponding genes of the TFs in that clique differ (Fig. 4a). For example, in the analysis of the interaction loops of enhancers and promoters that are shared by GM12878, K562, and MCF7 cells, a clique with CTCF-RAD21-SMC3-YY1-ZNF143-CTCFL-SIX5 TFs is shared by all these three cells. The expression levels of these TF corresponding genes differ (Fig. 4b). Figure 4b indicates that the expression levels of CTCF and CTCFL are dissimilar among the GM12878, K562, and MCF7 cells. In addition, Additional file 3: Figure S4 shows the distributions of these seven TF binding sites and loops of enhancers and promoters in hierarchical structures of chromatin in chromosome 19. The results demonstrate that the binding strength of seven TFs vary across loops in hierarchical structures.

We further analyze the methylation extent of the promoters of genes regulated by the clique of CTCF-RAD21-SMC3-YY1-ZNF143-CTCFL-SIX5 and shared by GM12878, K562, and MCF7 cells. The methylation extent is calculated for these promoters of the shared regulated genes by using cellMethy [42], which can quantify concordant methylation regions (See Methods). Analyzing the different methylation extents of the promoters of the shared regulated genes, we observe that the BCOR gene is shared by all these seven TFs (clique of CTCF-RAD21-SMC3-YY1-ZNF143-CTCFL-SIX5) in the GM12878, K562 and MCF7 cells. A recent publication has indicated that the BCOR gene has multiple methylated sites and can impact allele-specific gene expression and regulate the accessible chromatin within TADs in mice [43]. This finding suggests that the promoters of regulated genes by the same clique can vary in methylation extent and impact on cell-specific gene expression.

In the second pattern, although a clique is shared by different cell types, it regulates different genes. For example, the clique with GATA1-STAT2-ETS1-TAL1 is shared by K562, MCF7, and HUVEC (Fig. 4c). The Venn diagram of genes, which are regulated by this clique in K562, MCF7, and HUVEC, is shown in (Fig. 4c). Only five genes are shared by all these three cell lines. The expression levels of all these five genes differ from one another, as revealed by the heatmap in Fig. 4c. The shared genes between different cell types are also dissimilar. The discrepancies suggest that such clique may cross-talk with other TFs or cliques to synergistically regulate gene expression.

In the third situation, cliques in different cell types share part of the TFs. This pattern is the most common in different cell types. For example, four different cliques share TFs RAD21, YY1, and TFAP2A in K562, GM12878, HUVEC, and MCF7 (Fig. 4d). The expression levels of these shared TFs differ in the four cell types and labeled in green color adjacent to the gene name. Moreover, these shared TFs tend to work together with other TFs in different cell types. Another example is the cliques with SIX5, NRF1, and TFAP2A, which are shared by all four cell lines (Fig. 4d). The expression of these three TFs also differs among the four cell lines.

These results indicate that the synergistic cooperation between TFs is dynamic in different cell types. Several TFs can have conserved cooperation in different cell types and be regulated by other cell-specific TFs. For a given TF, numerous cooperation patterns may occur in different cell types and can determine the cell-specific gene expression and 3D organization of chromatin.

### Biclustering analysis of cliques and their regulated genes

To further investigate the possible cross-talk between different cliques, we apply the biclustering method to group cliques and genes simultaneously. We adopt the 51 cliques and 1223 regulated genes of K562 as an example and apply the biclustering method fabia [44] to group cliques and genes into 10 classes simultaneously (Additional file 5). The iteration times are set to 10,000.

**Fig. 5:**
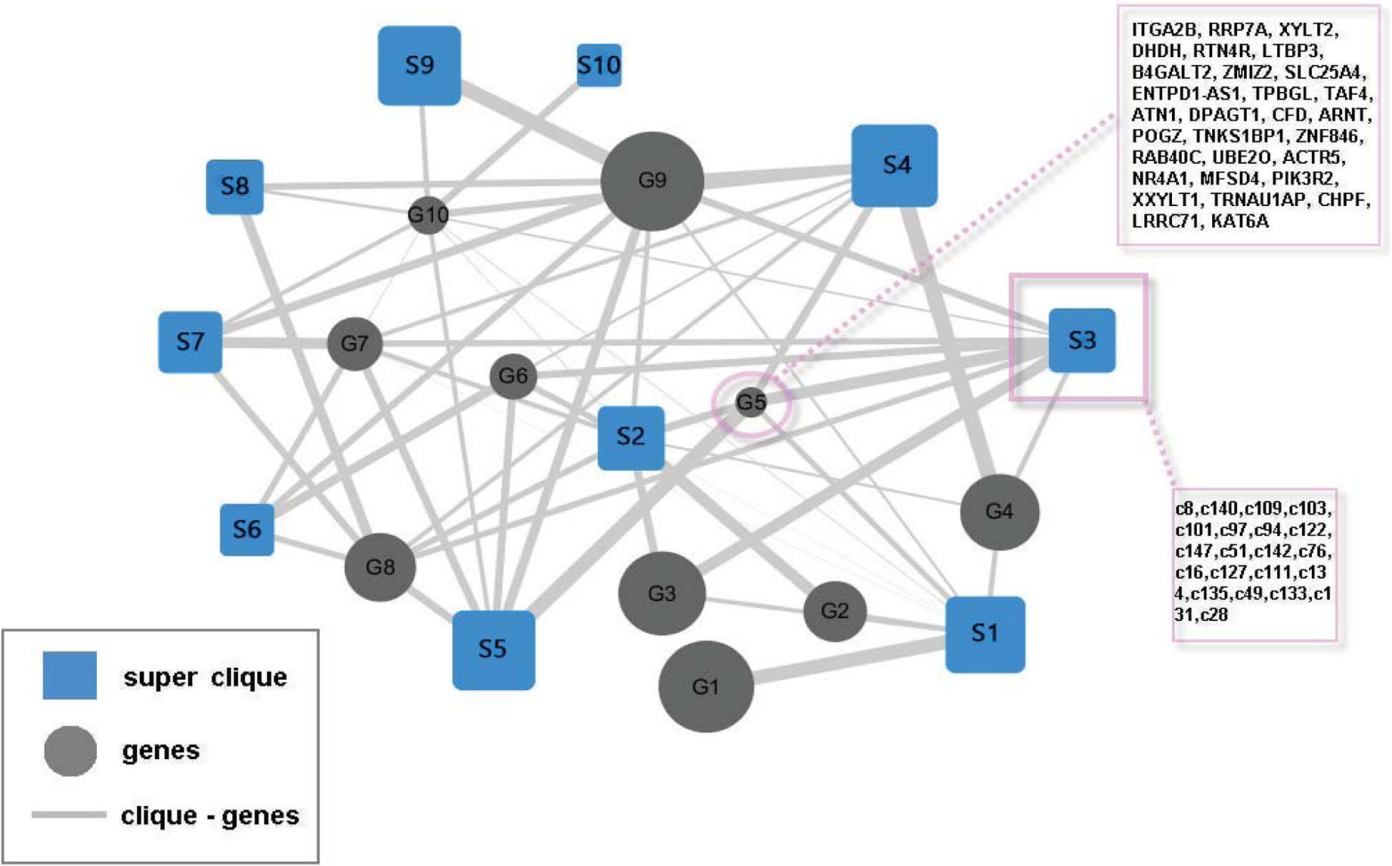
Biclustering analysis of cliques and their regulated genes. The line strength of interactions between supercliques and gene groups is proportional to the numbers between them

The 10 groups of bipartite networks of cliques and genes are given (Fig. 5). The specific clique and gene list for each superclique (Si, represented by a blue square) and gene group (Gi, represented by a gray circle) are listed in Additional file 5. Figure 5 shows that one gene group is regulated by more than one superclique, and one superclique can regulate more than one gene group. The regulated strength differs between the superclique and the gene group. Cliques can form supercliques to synergistically regulate the expression of a set of genes. The results also suggest that the regulation of genes at the clique level is hierarchical and dynamic.

## Discussion

In this study, we develop the first method to investigate the hierarchy and dynamics of TF cooperation by integrating various data, such as ChIA-PET, ChIP-Seq, and PPI. Using the high resolution and throughput data of ChIA-PET and ChIP-Seq, our method can generate a network with TFs from 3D and 1D chromosome information and calculate the TFs’ hierarchical levels, such as whole-cell networks, communities, cliques, and supercliques. The TFs in enhancers and promoters have intensive cross-talks and can form a hierarchical structure to dynamically regulate gene expression. For example in the GM12878 cell, the TFs can be grouped into six levels. A given TF can participate in several communities, and a community can contain several TFs across different communities. One TF can synergistically work together with other TFs from different hierarchical levels and communities to play multiple roles in gene expressions. The cooperation of TFs can form the maximal cliques, which may shape the specific gene expression of cells. TFs in a clique tend to participate in several hierarchical levels and communities. The genes in the same clique tend to be co-expressed and synergistically co-regulated. The cooperation of given TFs in cliques can have three patterns in different cell types. In one pattern, a clique is shared by different cell types, and the regulated genes of such clique are nearly the same across different cell types. However, the expression of TFs in such clique can differ among the cell types involved. The sites and bindinpromoters is a hierarchical and strength of TFs in linear DNA are dissimilar in different cell types. The methylation extent and sites for the regulated genes also vary. This pattern demonstrates that beside the cooperation of TFs, the expression, binding strength, and sites of the TFs in linear DNA can impact gene expression and chromatin structure. In the second pattern, cells share the same clique, but the regulated genes differ. In the third pattern, cliques in different cell types share a part of TFs, and the regulated genes are dissimilar. Some cooperative interactions of TFs tend to be conserved in different cell types and work together with other TFs to form cliques to synergistically regulate and shape cell-specific gene expression. This occurrence shows the dynamic proprieties of TFs in cooperation. All these three patterns imply that the TF cooperations are dynamic and can be regulated by epigenetic factors, such as other TFs and DNA methylation. Biclustering analysis of the cliques and their regulated genes indicate that a clique can function together with other cliques to form a superclique to synergistically regulate gene expression. This observation further suggests the presence of a hierarchical propriety among the TFs in cooperation. All these results demonstrate that the interaction of enhancers and promoters is a hierarchical and dynamics complex process with cooperative interactions of different TFs synergistically regulating gene expression and chromatin structure. These results also indicate that the regulation of chromatin 3D organization and gene expression is a complex process associated with the hierarchical and dynamic prosperities of TFs. Cell type-specific chromatin 3D organization and gene expression can be achieved on the basis of the dynamic feature of TF synergistic potential functions in different cell types.

## Conclusions

This study provides a systematic approach to study the hierarchy and dynamics of TF cooperation in chromatin 3D and 1D space by using various data, such as the ChIA-PET, ChIP-Seq, and PPI data. The technique will pave the way toward research on chromatin 3D layer architecture mediated by TF cooperation through the hierarchical and dynamic properties of TFs. Such investigation can improve our understanding of the regulation of gene expression and the basis of the 3D chromosome structure.

## Methods

### Datasets

The raw ChIA-PET sequence datasets for the K562, GM12878, MCF7, and HUVEC cell lines are downloaded from the Gene Expression Omnibus (GEO) databases [45]. The specific accession ID for each dataset of a given cell line is listed in Additional file 1.

The chromatin states for K562, GM12878, and HUVEC are downloaded from the ENCODE project at UCSC [46] (Additional file 1). The chromatin states for MCF7 are downloaded from the GEO database (GSE57498) [47]. The RNA-Seq datasets for K562, GM12878, and MCF7 are downloaded from the ENCODE project at the UCSC (Additional file 1). Five replicates of the RNA-Seq datasets for K562 and GM12878 are adopted. The RNA-Seq data for HUVEC are downloaded from the GEO database (GSE103672) [48]. The TAD structure data of GM12878, K562, and HUVEC are downloaded from the dataset of GSE635259 that is deposited in the GEO database [45]. The resolution of the TAD structure is 50 kb in this study. The reference PPI is constructed using STRING [49] and BioGRID [50]. The non-redundant peaks of 237 TFs are downloaded from the ReMap database [22], and all these peaks are calculated using ChIP-Seq datasets [22]. A full list with 2684 PWMs for 980 TFs is collected from a previous publication [26]. The bisulfite-sequencing (RRBS) methylation data of K562, GM12878, and MCF7 are downloaded from ENCODE at UCSC [51] (Additional file 1). The methylation site is calculated by using Bismark [52], and the methylation extent is calculated for these shared promoters by using cellMethy [42]. All the specific websites of the datasets used in this study are found in Additional file 1.

### Data processing

The DNA–DNA contacts are computed using our developed method CHIA-PET Tool [53], and the procedures for processing of ChIA-PET sequence data are briefly given as below. First, the raw ChIA-PET sequence datasets for a given cell are linker filtered on the basis of the linker information. Then, only filtered reads (non-chimeric PETs) are mapped to the human reference genome hg19 and classified as non-mappable PETs, uniquely mapped PETs, and multi-mapped PETs. All uniquely mapped PETs with a 1–2 base pair difference are merged and classified as self-ligation PETs and inter-ligation PETs. Self-ligation PETs are used for peak calling, where inter-ligation PETs are adopted to compute long-range DNA–DNA interactions. All interactions with more than five tags are used for downstream analysis. For the DNA–DNA contacts of the four cell lines of the ChIA-PET datasets, each anchor in two DNA–DNA contacts is annotated using the chromatin states of the corresponding cell line. Only the DNA–DNA contacts with concurrent enhancer and promoter annotations are retained and taken as the final 3D interaction datasets. Because each antibody can only capture a fraction of DNA–DNA contacts of the whole cell, the calculated DNA–DNA contacts are combined and the duplicate contacts are merged. Then, the resulted DNA–DNA contacts are taken as the final DNA–DNA contacts for a given cell.

### Network construction

To systematically construct the TF network, we calculate for the co-localization of TFs by using their binding sites on the basis of the ChIP-Seq and ChIA-PET data. We also build the reference PPI on the basis of STRING [49] and BioGRID [50].

For linear DNA sequences of all 237 TF ChIP-Seq datasets, the binding sites of each TF pair are used to calculate the significance overlap using IntervalStats [27]. The *p*-values of each peak of a given TF are calculated against the peaks of the other TFs. The ratio of the significant overlapped peaks, in which their *p*-values are smaller than 0.05 to all peaks, are computed as the similarity of two TFs. Each TF is considered as a query set only once with other TFs as reference. An asymmetric matrix with similarities between all 237 TFs, which are 55,932 pairs of TFs, is calculated and named as 1D matrix in the following study.

The promoter and enhancer sequences, which are annotated by chromatin states, are used to be scanned the enriched TFs by using a given TF PWM. A full list with 2684 PWMs for 980 TFs is collected from a previous publication [26] and used for TF enrichment analysis as follows. The enriched TFs in each promoter are calculated using the PASTAA [54] and taken as the candidate TFs that can bind to the promoters. The enriched TFs in each enhancer are then calculated using the FIMO [55] and taken as the candidate TFs that can bind to the enhancers. The number of selected TFs is related to the choice of *p*-values of PASTAA and FIMO. A small *p*-value can reduce the false positive rate, but the number of selected TFs also decreases. To balance the *p*-value for PASTAA and FIMO and the number of selected TFs of the four cell lines, we optimize the *p*-value on the basis of the Jaccard similarity of the four cell lines, with a range from 10^− 7^ to 10^− 13^ at a multiplication interval of 10^− 1^. Then, the inflection point is calculated (Methods in Additional file 1). In this study, the *p*-value is set to 10^− 10^. The final TF list is the intersection of the promoter and enhancer enrichment TF lists.

The reference PPI is constructed by using the STRING [49] and BioGRID [50] databases. For the STRING database, the human PPIs with scores of more than 400 are retained. For the BioGRID database, the human PPIs with experimental validated information are retained. Then, the PPIs shared by both databases are taken as the final reference PPI. By using the above final TF list for a given cell that is enriched in promoters and enhancers, the PPIs, including the proteins in the final TF list, are selected on the basis of the final reference PPI. These PPIs for the given TF list of cells are taken as the 3D TF interactions. Then, we obtain the TF interaction matrix for ChIA-PET datasets for a given cell. This TF interaction matrix is named as 3D TF interaction matrix. The number of TFs in the 3D TF interaction matrix for the four cell lines are given in Table 1(Column 3D TFs in Table 1).

**Table 1:** Cell line-specific TFs

Cell lines	3D TFs	3D–1D fusion TFs
K562	423	72
GM12878	428	73
HUVEC	394	58
MCF7	410	70

### Similarity matrix fusion

The above 1D and 3D matrices represent the TF interactions in a particular viewpoint. We combine these two matrices to obtain a comprehensive TF interaction network for each cell. To fuse the similarity matrix of both 1D and 3D matrices, we calculate the shared TFs between 1D and 3D matrices. The result is shown in Table 1. The sub-matrix with these shared TFs is extracted for the 1D and 3D matrices, respectively. Then, the sub-matrix is fused by using the similarity network fusion [28] algorithm, a network-based heterogeneous data integration method. The fused matrix with TFs shared by the 1D and 3D matrices is taken as the final TF network and used in the following analysis.

### Hierarchical network

The hierarchical structure is an important feature of society networks [56] and genetic regulatory networks [57]. The hierarchical structure of a TF network is analyzed with the hierarchical score maximization method [38]. The corrected hierarchical score [38] is used to quantify the level of TF network, and the number of levels is optimized from 2 to 8 with an interval of 1. The analysis is performed to determine whether a hierarchical structure that can regulate the 3D chromosome structure and gene regulation exists in the TF network. The hierarchical organization of the TF network can reveal the synergistic mechanism and offer new insights into the 3D chromosome structure and gene regulation.

### Network community analysis

Because one protein may participate in several communities in a cell, the community detection of a TF network should include potentially overlapping TF complexes. In this work, the ClusterONE method [29] is applied to calculate for the communities with overlapping TF complexes. ClusterONE proposes a metric of cohesiveness score to detect the densely connected TFs through different seeds for a given network.

### Maximal clique analysis

To understand the local structure property of the TF network, we calculate the maximal clique by the MClique method in Cytoscape [41]. The number of TFs in maximal cliques ranges from 3 to 10. To further obtain the synergistic working cliques, this study represents the regulated gene for a given clique as 1 and the other non-regulated genes as 0. Then, the biclustering method fabia (factor analysis for bicluster acquisition) [44] is used to group cliques and genes simultaneously. Fabia is a factor analysis method based on the multiplicative model. The grouped cliques are named as a superclique. A graph with supercliques and gene groups is generated using Cytoscape.

## Additional files

Additional file 1: All datasets used in this study (ChIA-PET, Chromatin states, TAD structure, and bisulfite-sequencing [RRBS] RNA-Seq) with accession codes and Parameter selection. To balance the *p*-value for PASTAA and FIMO and the number of selected TFs of the four cell lines, we optimize the *p*-value on the basis of the Jaccard similarity of the four cell lines, with a range from 10^− 7^ to 10^− 13^ at a multiplication interval of 10^− 1^. Then, the inflection point is calculated. (DOCX 18 kb)

Additional file 2: Illustration of the fused network communities of TFs and the overlapping communities of GM12878 cell line. Nine groups are calculated, and the specific list of TFs in each community is listed. (XLSX 10 kb)

Additional file 3: Supporting figures. **Figure S1.** Hierarchical structures of the GM12878 TFs network. **Figure S2.** Percentage of TFs from different levels in the phylostrata. **Figure S3.** Violin plots of Spearman’s rank correlation coefficient for TF expressions in the same clique or not in four cell lines. **Figure S4.** Distribution of ZNF143-YY1-SMC3-SIX5-RAD21-CTCF-CTCFL seven TF binding sites and loops of enhancer and promoters in hierarchical structures of chromatin in chromosome 19 of GM12878 cell line. **Figure S5.** Parameter selection in motif scanning. (PDF 687 kb)

Additional file 4: Calculation for the maximal cliques for the fused matrix of TFs in different cell types, the specific TF in each maximal clique for the four cell lines is listed. (XLSX 12 kb)

Additional file 5: The biclustering results of cliques and genes. We adopt the 51 cliques and 1223 regulated genes of K562 as an example and apply the biclustering method fabia to group cliques and genes into 10 classes. The specific clique and gene list for each superclique (Si, represented by a blue square) and gene group (Gi, represented by a gray circle) are listed. (XLSX 22 kb)

## Abbreviations

1D: Linear DNA
3D: 3 dimension
ChIA-PET: Chromatin Interaction Analysis by Paired-End Tag Sequencing
ChIP-Seq: Chromatin Immunoprecipitation Sequencing
GM12878: B lymphocyte cell line
Hi-C: High-throughput chromosome conformation capture
HUVEC: Human umbilical vein endothelial cell line
K562: Human immortalized myelogenous leukemia line
MCF7: Human breast adenocarcinoma cell line
PPI: Protein-protein interaction
PWMs: Position weight matrices
TADs: Topologically associated domains
TFs: Transcription factors
