# Supplementary material for: Hierarchical cooperation of transcription factors from integration analysis of DNA sequences, ChIP-Seq and ChIA-PET data
Source: BMC Genomics. 2019 May 8;20(Suppl 3):296. doi: 10.1186/s12864-019-5535-2 (PMC7226942; doi:10.1186/s12864-019-5535-2)
Supplement: Supplementary file 1 — All datasets used in this study (ChIA-PET, Chromatin states, TAD structure, and bisulfite-sequencing [RRBS] RNA-Seq) with accession codes and Parameter selection. To balance the p-value for PASTAA and FIMO and the number of selected TFs of the four cell lines, we optimize the p-value on the basis of the Jaccard similarity of the four cell lines, with a range from 10− 7 to 10− 13 at a multiplication interval of 10− 1. Then, the inflection point is calculated. (DOCX 18 kb) [file 12864_2019_5535_MOESM1_ESM.docx]

**DATASET**

| **ChIA-PET datasets** | | | |
| --- | --- | --- | --- |
| **Cell line** | | **Accession No.** | **Antibodys** |
| K562 |  | GSE33664, GSE59395 | RNA PII |
| GM12878 | | GSE72816 | RNA PII |
| MCF7 |  | GSE33664 | RNAPII |
| HUVEC | | GSE41553 | RNAPII |
| **Chromatin states datasets** | | | |
| **Cell line** |  | **IDENTIFIER** | |
| K562/ GM12878/ HUVEC | [http://www.genome.ucsc.edu/cgi-bin/hgFileUi?db=hg19&g=wgEncod](http://www.genome.ucsc.edu/cgi-bin/hgFileUi?db=hg19&amp;g=wgEncod) eAwg Segmentation | | |
| MCF7 |  | GSE57498 | |
| **RNA-Seq datasets** | | | |
| **Cell line** |  | **IDENTIFIER** | |
| K562 | https:[//www.enc](http://www.encodeproject.org/search/)o[dep](http://www.encodeproject.org/search/)r[oject.org/search/?](http://www.encodeproject.org/search/) type=Experiment&biosample  _term_name=K562&assay_slims=Transcription&assay_title=total+RNA-seq | | |
| GM12878 | https:[//www.enc](http://www.encodeproject.org/search/?type=Experiment&amp;biosample_)o[dep](http://www.encodeproject.org/search/?type=Experiment&amp;biosample_)r[oject.org/search/?type=Experiment&biosample_](http://www.encodeproject.org/search/?type=Experiment&amp;biosample_)  term_name=GM12878&assay_title=total+RNA-seq | | |
| MCF7 | https:[//www.enc](http://www.encodeproject.org/)o[dep](http://www.encodeproject.org/)r[oject.org/](http://www.encodeproject.org/) search/?type=Experiment&biosample  _term_name=MCF-7&assay_title=total+RNA-seq | | |
| HUVEC |  | GSE103672 | |
| **PPI datasets** | | | |
| STRING V10 | | https://string-db.org |  |
| BioGRID 3.4.149 | | https://thebiogrid.org |  |
| **Bisulfite-sequencing (RRBS) methylation datasets** | | | |
| K562/GM12878/  MCF7 | [http://genome.ucsc.edu/cgi-bin/hgTrackUi?db=hg19&g=wgEncode](http://genome.ucsc.edu/cgi-bin/hgTrackUi?db=hg19&amp;g=wgEncode)  HaibMethylRrbs | | |

**METHODS**

To balance the *p*-values for PASTAA and FIMO and the number of selected TFs in GM12878, K562, MCF7 and HUVEC four cell lines, the *p*-value was optimized range from ${10}^{-7}$ and ${10}^{-13}$. We defined a parameter γ to evaluate the consistency of *p*-values.

$\Upsilon=\frac{1}{C_{m}^{2}}*\frac{1}{N}*\sum_{X_{i,j}} (X_{i}\cup X_{j})(1-\frac{X_{i}\cap X_{j}}{X_{i}\cup X_{j}})$ (1)

Here N represents the number of background TFs and N is 980 in this study. m represents the number of cell lines. $X_{i}$ and $X_{j}$ represent the corresponding number of TFs in cell lines of i and j respectively. The γ is plotted against with the p-values and is shown in the following Fig. S5. The inflection point is calculated and located at *p*-value of 10^-10^. So, 10^-10^ is selected as the final p-value.
