# Supplementary material for: Hierarchical cooperation of transcription factors from integration analysis of DNA sequences, ChIP-Seq and ChIA-PET data
Source: BMC Genomics. 2019 May 8;20(Suppl 3):296. doi: 10.1186/s12864-019-5535-2 (PMC7226942; doi:10.1186/s12864-019-5535-2)
Supplement: Supplementary file 3 — Supporting figures. Figure S1. Hierarchical structures of the GM12878 TFs network. Figure S2. Percentage of TFs from different levels in the phylostrata. Figure S3. Violin plots of Spearman’s rank correlation coefficient for TF expressions in the same clique or not in four cell lines. Figure S4. Distribution of ZNF143-YY1-SMC3-SIX5-RAD21-CTCF-CTCFL seven TF binding sites and loops of enhancer and promoters in hierarchical structures of chromatin in chromosome 19 of GM12878 cell line. Figure S5. Parameter selection in motif scanning. (PDF 687 kb) [file 12864_2019_5535_MOESM3_ESM.pdf]

a

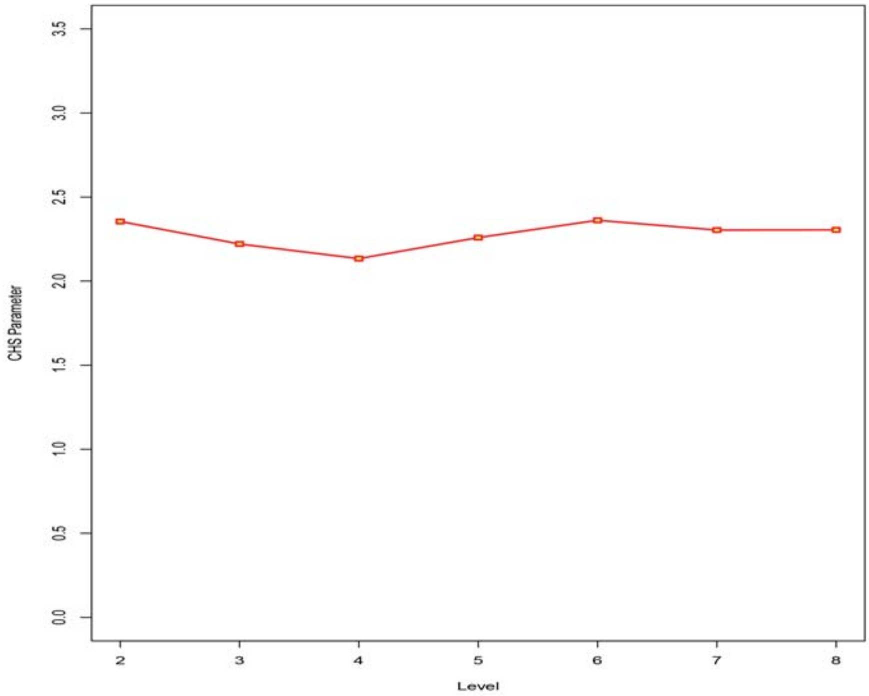

b

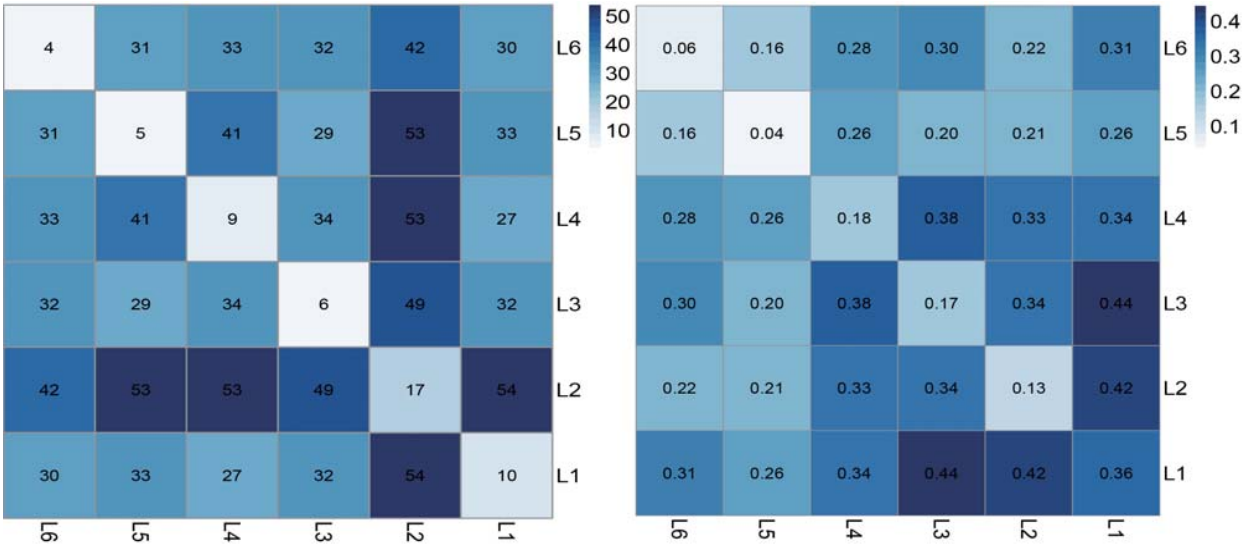

Fig. S1. Hierarchical structures of the GM12878 TFs network. (a) The corrected hierarchy score for different levels (b) Heatmaps for absolute number (left) and ratio (right) of links across different levels.

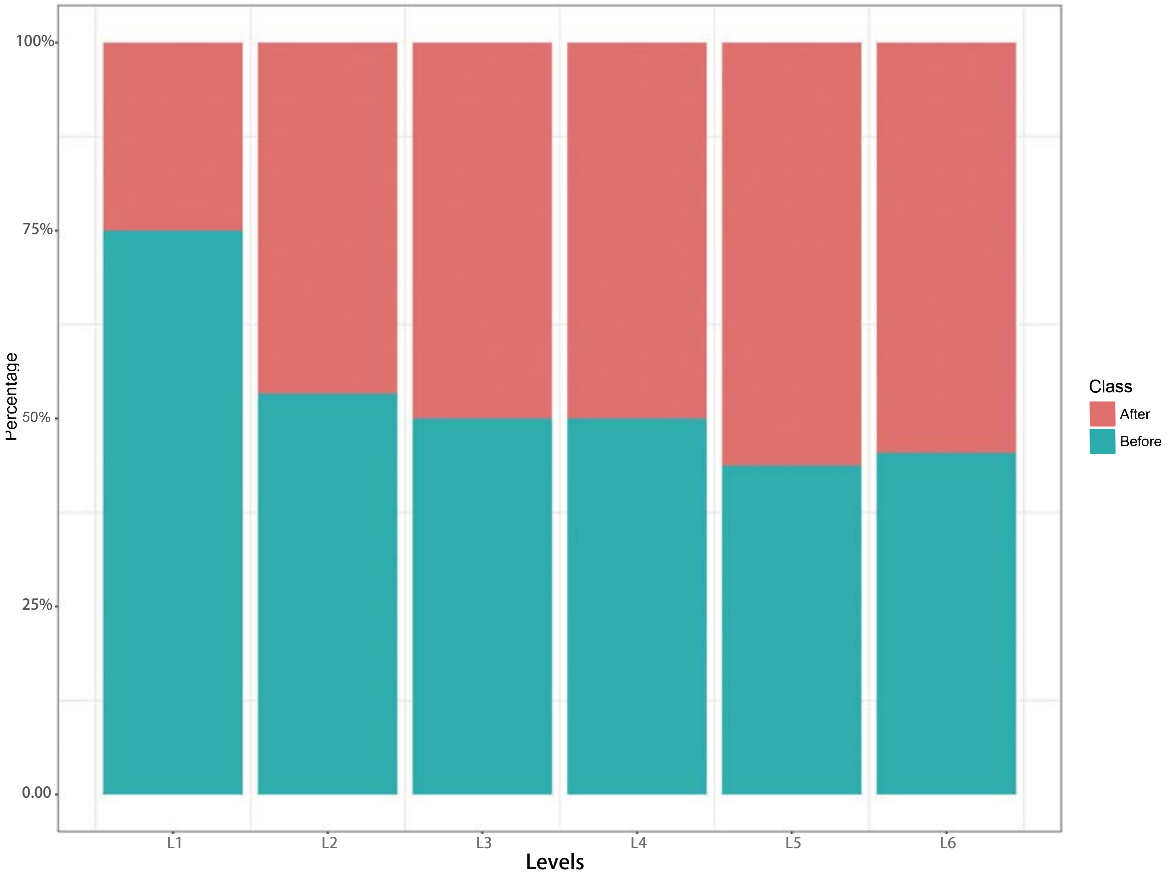

Fig. S2. Percentage of TFs from different levels in the phylostrata. Blue represents the TF that emerged before the Bil ateria phylostratum, and red represents the TF that emerged in and after the Bilateria phylostratum.

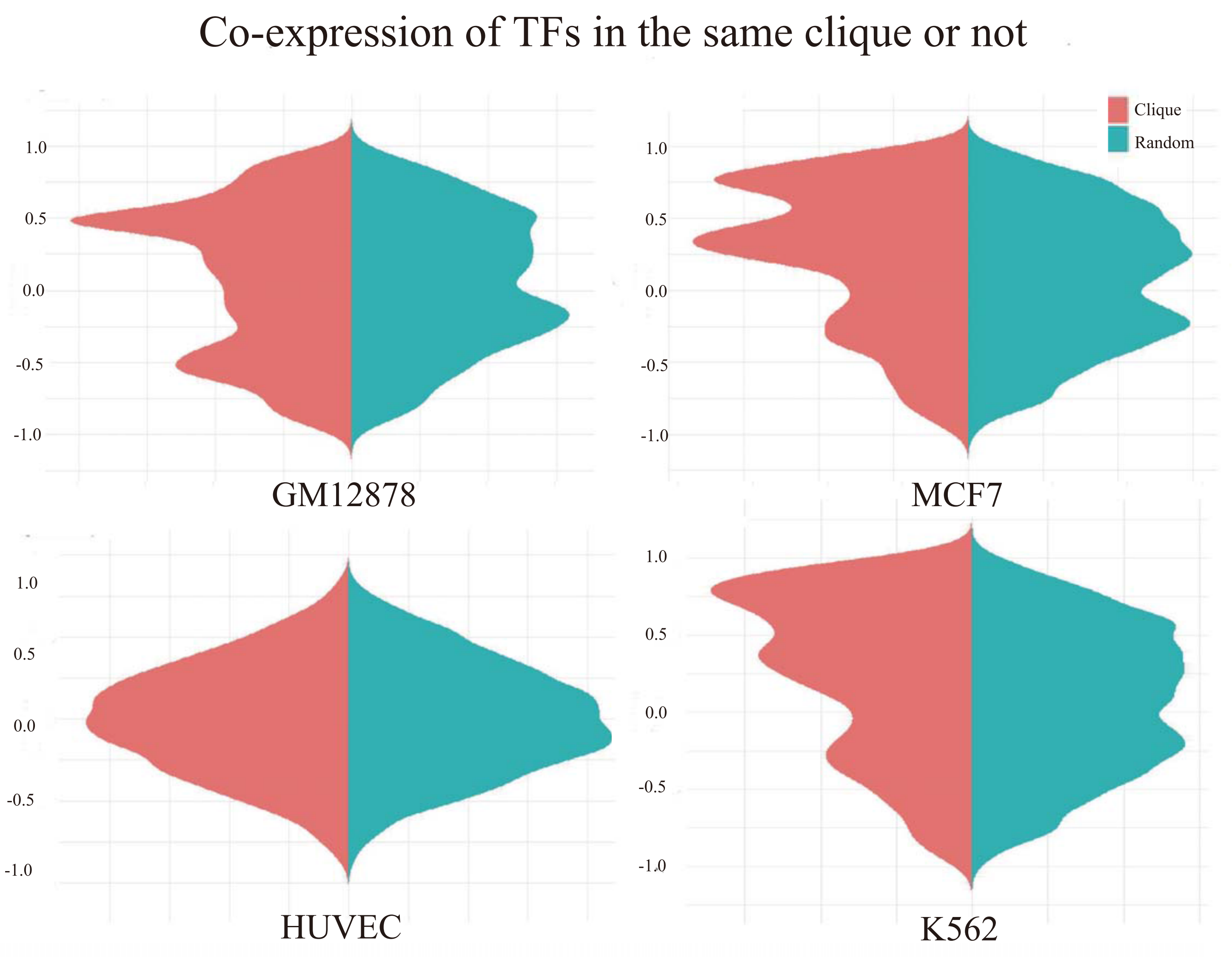

Fig. S3. Violin plots of Spearman's rank correlation coefficient for TF expressions in the same clique or not in four cell lines.

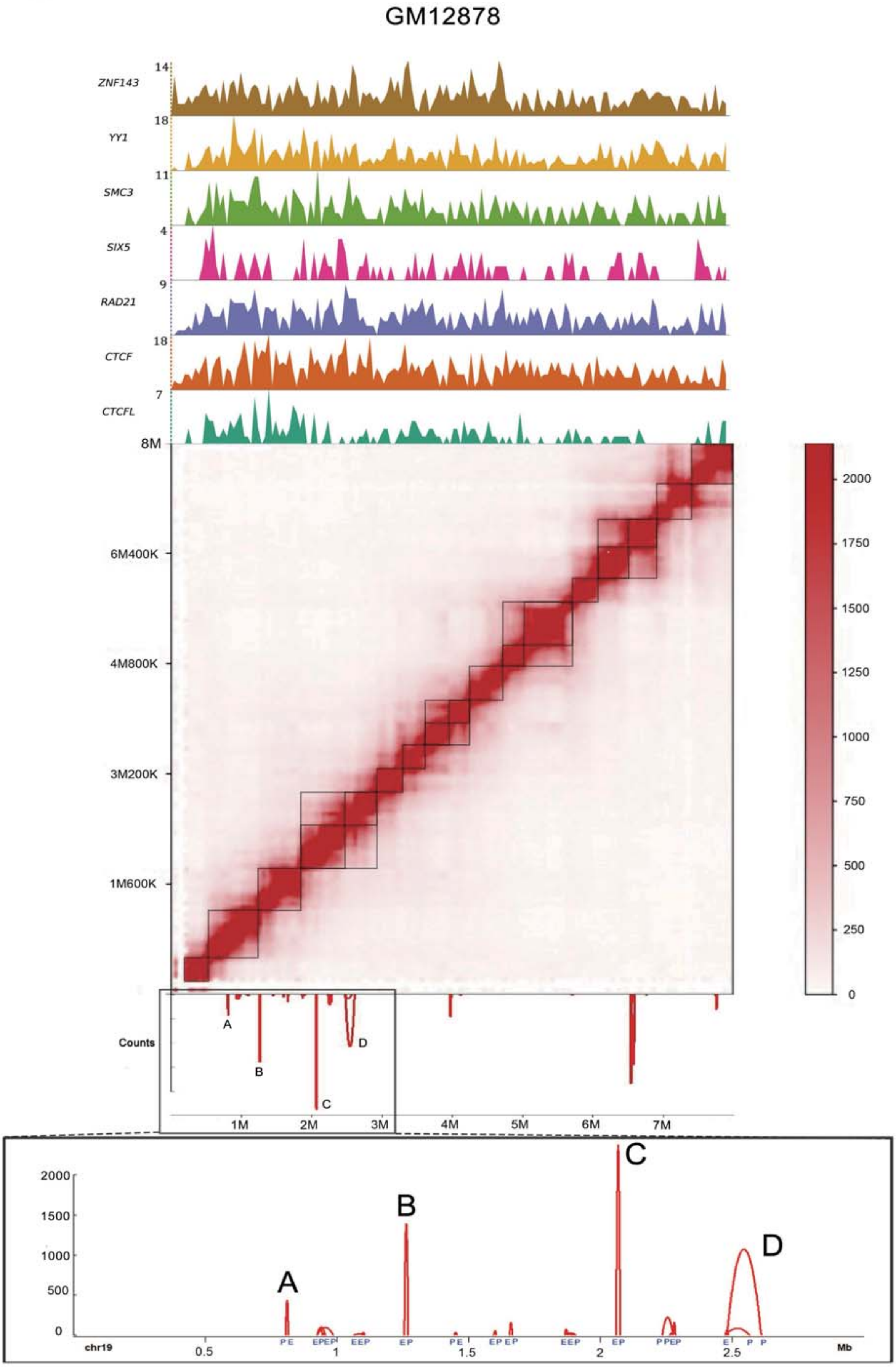

Fig. S4. Distribution of ZNF143-YY1-SMC3-SIX5-RAD21-CTCF-CTCFL seven TF binding sites and loops of enhancer and promoters in hierarchical structures of chromatin in chromosome 19 of GM12878 cell line.

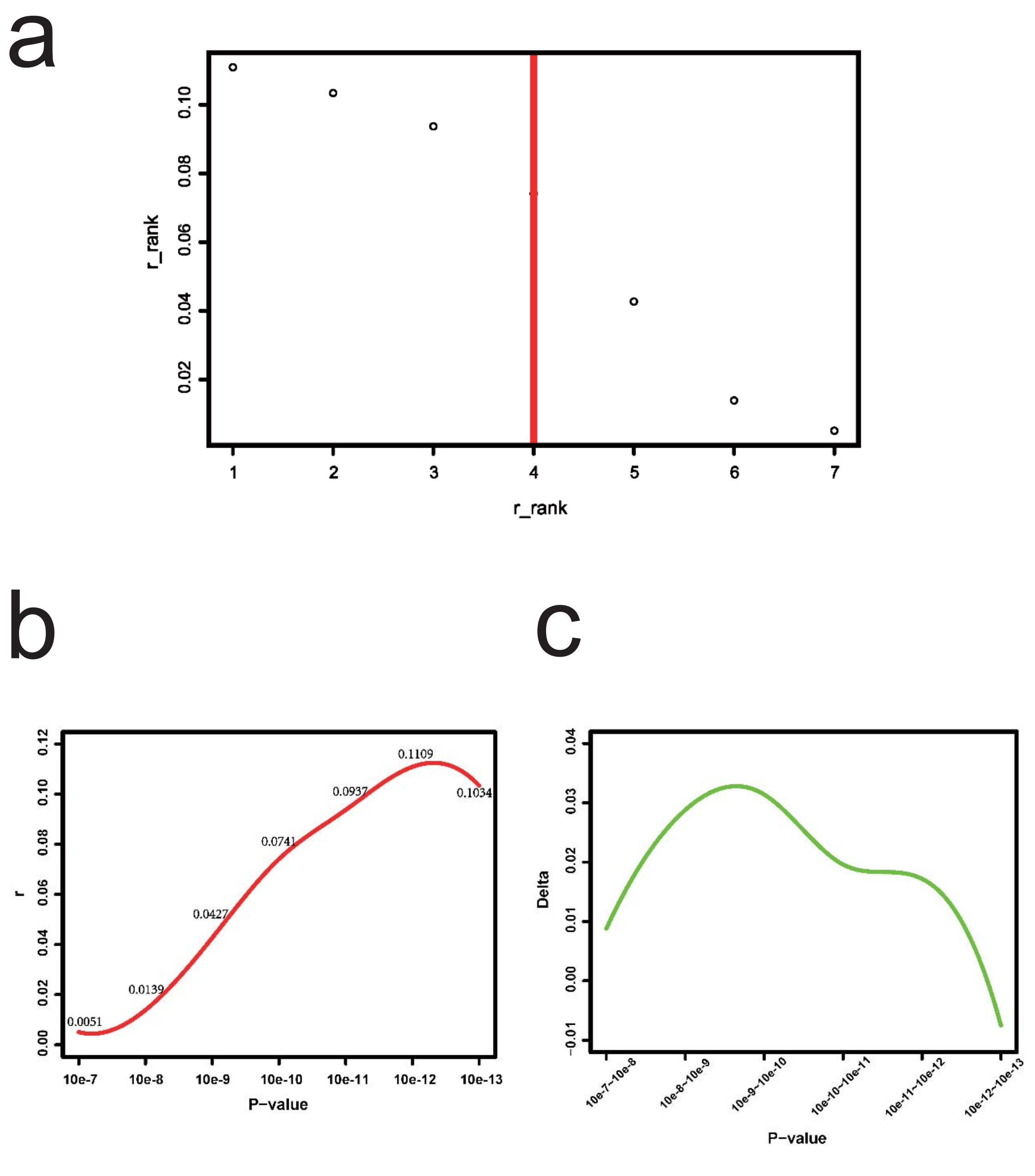

Fig. S5. Parameter selection in motif scanning. (a) The γ_rank of different P-values, the red vertical line located in P-value of 10-10; (b) The γ of different P-values; (c) The Δ between different P-values.
